# Bis(μ-*N*-acetyl-*N*-phenyl­glycinato-κ^2^ *O*:*O*′)bis­[dinitrato-κ^4^ *O*,*O*′-bis­(1,10-phenanthroline-κ^2^ *N*,*N*′)lanthanum(III)]

**DOI:** 10.1107/S1600536808038075

**Published:** 2008-12-13

**Authors:** Xiaonan Gao

**Affiliations:** a Department of Chemistry, East Tennessee State University, Johnson City, Tennessee, USA

## Abstract

In the title complex, [La_2_(C_10_H_10_NO_3_)_2_(NO_3_)_4_(C_12_H_8_N_2_)_4_], each La^III^ ion is ten-coordinated by four N atoms from two bidentate 1,10-phenanthroline ligands and by six O atoms, two from the *N*-acetyl-*N*-phenyl­glycinate ligands and four from two nitrate anions. Two La^III^ cations, which exhibit a distorted bicapped square-anti­prismatic coordination, are bridged by two *N*-acetyl-*N*-phenyl­glycinate ligands into a dimeric structure, generated by inversion symmetry. There is a π–π contact between the benzene rings [centroid–centroid distance = 3.409 (3) Å].

## Related literature

For related structures, see: Fu *et al.* (2004*a* ▶,*b* ▶).
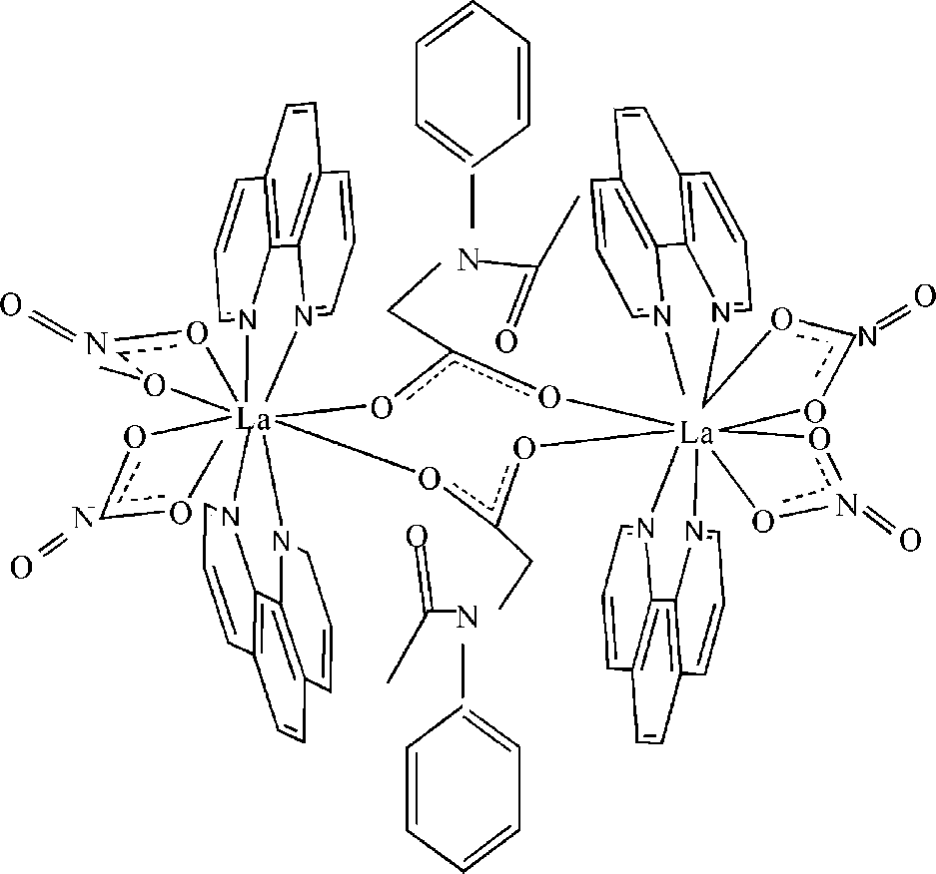


## Experimental

### 

#### Crystal data

[La_2_(C_10_H_10_NO_3_)_2_(NO_3_)_4_(C_12_H_8_N_2_)_4_]
*M* *_r_* = 1631.06
Monoclinic, 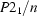

*a* = 14.0891 (7) Å
*b* = 13.7610 (7) Å
*c* = 16.9962 (9) Å
β = 100.121 (1)°
*V* = 3243.9 (3) Å^3^
*Z* = 2
Mo *K*α radiation
μ = 1.39 mm^−1^
*T* = 296 (2) K
0.45 × 0.43 × 0.40 mm

#### Data collection

Bruker SMART CCD area-detector diffractometer
Absorption correction: multi-scan (*SADABS*; Bruker, 1997 ▶) *T*_min_ = 0.574, *T*_max_ = 0.543 (expected range = 0.607–0.574)
16038 measured reflections
5719 independent reflections
4467 reflections with *I* > 2σ(*I*)
*R*_int_ = 0.031

#### Refinement

*R*[*F* ^2^ > 2σ(*F* ^2^)] = 0.025
*wR*(*F* ^2^) = 0.059
*S* = 1.06
5719 reflections
461 parameters
H-atom parameters constrained
Δρ_max_ = 0.70 e Å^−3^
Δρ_min_ = −0.42 e Å^−3^

### 

Data collection: *SMART* (Bruker, 1997 ▶); cell refinement: *SAINT* (Bruker, 1997 ▶); data reduction: *SAINT*; program(s) used to solve structure: *SHELXS97* (Sheldrick, 2008 ▶); program(s) used to refine structure: *SHELXL97* (Sheldrick, 2008 ▶); molecular graphics: *SHELXTL* (Sheldrick, 2008 ▶); software used to prepare material for publication: *SHELXTL*.

## Supplementary Material

Crystal structure: contains datablocks I, global. DOI: 10.1107/S1600536808038075/hk2563sup1.cif

Structure factors: contains datablocks I. DOI: 10.1107/S1600536808038075/hk2563Isup2.hkl

Additional supplementary materials:  crystallographic information; 3D view; checkCIF report

## Figures and Tables

**Table 1:** Selected bond angle (°)

O1—La1—O4	74.74 (7)

**Fig. 1.**
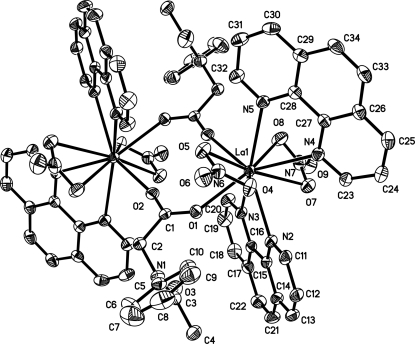
The molecular structure of the title molecule, with the atom-numbering scheme. Displacement ellipsoids are drawn at the 30% probability level [symmetry code: (i) 2 - x, 2 - y, 2 - z].

## supplementary crystallographic information

### Comment

*N*-acetyl-*N*-phenylglycinate should be a very useful multidentate ligand and may act as bridge ligand due to coordination of its acetyl O and carboxylate O atoms. A few multinuclear complexes have been synthesized with monodeprotonated *N*-acetyl-*N*-phenylglycinate as bridge ligand (Fu *et al.* 2004*a*,*b*). We were interested in synthesizing multinuclear complexes with they as bridge ligands, and obtained the title dinuclear complex. We reported herein its crystal structure.

The coordination structure of the title complex is shown in Fig. 1. In the dinuclear complex, each La^III^ ion is ten-coordinated by atoms N2, N3, N4 and N5 from two 1,10-phenanthroline (L2) ligands, atoms O4, O5, O7 and O8 from two nitrate anions (L3), atoms O1, O2^i^ [symmetry code: (i) 2 - x, 2 - y, 2 - z] from carboxylate groups of two *N*-acetyl-*N*-phenylglycinate (L1) ligands. The coordination sphere around La is a distorted bicapped square-antiprism, with the capping positions occupied by atoms O5 of nitrate anion and O7 of another nitrate anion. The coordinated L1 ligand bonds to two La *via* two O atoms of carboxylate group, thus acting as a bridge. The overall complex has inversion symmetry. The π–π contact between the benzene rings, Cg1···Cg1^ii^ [symmetry code: (ii) x - 1, y, z - 1, where Cg1 is the centroid of the ring (C14–C17/C21/C22)] may stabilize the structure, with centroid–centroid distance of 3.409 (3) Å.

### Experimental

La(NO_3_)_3_.nH_2_O (1 mmol) and 1,10-phenanthroline (1 mmol) were dissolved in anhydrous ethanol (20 ml). To this solution, an aqueous mixture (10 ml) of *N*-acetyl-*N*-phenylglycinate (1 mmol) and NaOH (1 mmol) was added dropwise. The mixture was stirred for 2 h. The large yellow crystals were obtained after the solution had been allowed to stand at room temperature for three weeks.

### Refinement

H atoms were positioned geometrically, with C—H = 0.93, 0.97 and 0.96 Å for aromatic, methylene and methyl H, respectively, and constrained to ride on their parent atoms with U_iso_(H) = xU_eq_(C), where x = 1.5 for methyl H and x = 1.2 for all other H atoms.

### Crystal data

**Table:**

[La_2_(C_10_H_10_NO_3_)_2_(NO_3_)_4_(C_12_H_8_N_2_)_4_]	*F*(000) = 1632
*M**_r_* = 1631.06	*D*_x_ = 1.670 Mg m^−^^3^
Monoclinic, *P*2_1_/*n*	Mo *K*α radiation, λ = 0.71073 Å
Hall symbol: -P 2yn	Cell parameters from 6044 reflections
*a* = 14.0891 (7) Å	θ = 2.3–27.5°
*b* = 13.7610 (7) Å	µ = 1.39 mm^−^^1^
*c* = 16.9962 (9) Å	*T* = 296 K
β = 100.121 (1)°	Needle, yellow
*V* = 3243.9 (3) Å^3^	0.45 × 0.43 × 0.40 mm
*Z* = 2	

### Data collection

**Table:**

Bruker SMART CCD area-detector diffractometer	5719 independent reflections
Radiation source: fine-focus sealed tube	4467 reflections with *I* > 2σ(*I*)
graphite	*R*_int_ = 0.031
φ and ω scans	θ_max_ = 25.0°, θ_min_ = 1.9°
Absorption correction: multi-scan (*SADABS*; Bruker, 1997)	*h* = −15→16
*T*_min_ = 0.574, *T*_max_ = 0.543	*k* = −16→12
16038 measured reflections	*l* = −20→15

### Refinement

**Table:**

Refinement on *F*^2^	Primary atom site location: structure-invariant direct methods
Least-squares matrix: full	Secondary atom site location: difference Fourier map
*R*[*F*^2^ > 2σ(*F*^2^)] = 0.025	Hydrogen site location: inferred from neighbouring sites
*wR*(*F*^2^) = 0.059	H-atom parameters constrained
*S* = 1.06	*w* = 1/[σ^2^(*F*_o_^2^) + (0.0228*P*)^2^ + 0.722*P*] where *P* = (*F*_o_^2^ + 2*F*_c_^2^)/3
5719 reflections	(Δ/σ)_max_ = 0.002
461 parameters	Δρ_max_ = 0.70 e Å^−^^3^
0 restraints	Δρ_min_ = −0.42 e Å^−^^3^

### Special details

**Table:**

Geometry. All e.s.d.'s (except the e.s.d. in the dihedral angle between two l.s. planes) are estimated using the full covariance matrix. The cell e.s.d.'s are taken into account individually in the estimation of e.s.d.'s in distances, angles and torsion angles; correlations between e.s.d.'s in cell parameters are only used when they are defined by crystal symmetry. An approximate (isotropic) treatment of cell e.s.d.'s is used for estimating e.s.d.'s involving l.s. planes.
Refinement. Refinement of *F*^2^ against ALL reflections. The weighted *R*-factor *wR* and goodness of fit *S* are based on *F*^2^, conventional *R*-factors *R* are based on *F*, with *F* set to zero for negative *F*^2^. The threshold expression of *F*^2^ > σ(*F*^2^) is used only for calculating *R*-factors(gt) *etc*. and is not relevant to the choice of reflections for refinement. *R*-factors based on *F*^2^ are statistically about twice as large as those based on *F*, and *R*- factors based on ALL data will be even larger.

### Fractional atomic coordinates and isotropic or equivalent isotropic displacement parameters (Å^2^)

**Table:**

	*x*	*y*	*z*	*U*_iso_*/*U*_eq_	
La1	0.845573 (12)	0.870851 (12)	0.945459 (10)	0.02900 (6)	
N1	1.13333 (19)	0.8679 (2)	0.79326 (15)	0.0430 (7)	
N2	0.90459 (18)	0.70723 (18)	0.87965 (14)	0.0359 (6)	
N3	0.99585 (18)	0.77290 (18)	1.02680 (15)	0.0367 (6)	
N4	0.66037 (18)	0.79638 (19)	0.89767 (15)	0.0379 (6)	
N5	0.68663 (18)	0.98664 (18)	0.94152 (15)	0.0375 (6)	
N6	0.80835 (19)	0.9726 (2)	0.78155 (17)	0.0428 (7)	
N7	0.8123 (2)	0.7497 (2)	1.08954 (18)	0.0495 (8)	
O1	0.99417 (15)	0.90185 (15)	0.88858 (12)	0.0408 (5)	
O2	1.09455 (15)	0.99913 (16)	0.96794 (12)	0.0415 (5)	
O3	1.20355 (19)	0.77683 (19)	0.89687 (15)	0.0648 (7)	
O4	0.78752 (16)	0.88449 (16)	0.79238 (13)	0.0477 (6)	
O5	0.83297 (17)	1.02339 (17)	0.84285 (14)	0.0539 (6)	
O6	0.80359 (19)	1.00540 (19)	0.71402 (15)	0.0617 (7)	
O7	0.80680 (17)	0.70496 (17)	1.02473 (14)	0.0513 (6)	
O8	0.8016 (2)	0.83975 (18)	1.08626 (14)	0.0616 (7)	
O9	0.8312 (2)	0.7069 (2)	1.15385 (16)	0.0747 (8)	
C1	1.0698 (2)	0.9493 (2)	0.90545 (18)	0.0324 (7)	
C2	1.1388 (2)	0.9516 (2)	0.84616 (19)	0.0445 (8)	
H2A	1.1260	1.0097	0.8138	0.053*	
H2B	1.2041	0.9565	0.8757	0.053*	
C3	1.1680 (3)	0.7818 (3)	0.8261 (2)	0.0476 (9)	
C4	1.1634 (3)	0.6951 (3)	0.7714 (2)	0.0578 (10)	
H4A	1.2042	0.7060	0.7326	0.087*	
H4B	1.0981	0.6858	0.7445	0.087*	
H4C	1.1847	0.6382	0.8022	0.087*	
C5	1.0850 (3)	0.8778 (2)	0.7122 (2)	0.0453 (8)	
C6	1.1368 (3)	0.9001 (3)	0.6535 (2)	0.0712 (12)	
H6	1.2029	0.9109	0.6664	0.085*	
C7	1.0902 (4)	0.9063 (4)	0.5747 (3)	0.0892 (16)	
H7	1.1254	0.9197	0.5345	0.107*	
C8	0.9928 (4)	0.8930 (3)	0.5559 (3)	0.0789 (14)	
H8	0.9618	0.8967	0.5030	0.095*	
C9	0.9415 (3)	0.8743 (3)	0.6144 (3)	0.0702 (12)	
H9	0.8749	0.8671	0.6016	0.084*	
C10	0.9867 (3)	0.8656 (3)	0.6927 (2)	0.0560 (10)	
H10	0.9510	0.8516	0.7324	0.067*	
C11	0.8644 (2)	0.6758 (2)	0.80770 (19)	0.0415 (8)	
H11	0.8133	0.7113	0.7796	0.050*	
C12	0.8938 (3)	0.5933 (3)	0.7716 (2)	0.0478 (9)	
H12	0.8634	0.5748	0.7208	0.057*	
C13	0.9677 (3)	0.5402 (3)	0.8120 (2)	0.0524 (10)	
H13	0.9877	0.4840	0.7894	0.063*	
C14	1.0140 (2)	0.5703 (2)	0.8879 (2)	0.0427 (8)	
C15	0.9805 (2)	0.6557 (2)	0.91969 (18)	0.0345 (7)	
C16	1.0281 (2)	0.6891 (2)	0.99698 (18)	0.0346 (7)	
C17	1.1050 (2)	0.6354 (2)	1.0390 (2)	0.0451 (8)	
C18	1.1482 (3)	0.6695 (3)	1.1146 (2)	0.0540 (10)	
H18	1.1990	0.6353	1.1446	0.065*	
C19	1.1157 (3)	0.7525 (3)	1.1438 (2)	0.0579 (10)	
H19	1.1435	0.7754	1.1941	0.069*	
C20	1.0401 (2)	0.8028 (3)	1.0977 (2)	0.0481 (9)	
H20	1.0197	0.8606	1.1178	0.058*	
C21	1.0924 (3)	0.5175 (3)	0.9327 (2)	0.0571 (10)	
H21	1.1138	0.4610	0.9114	0.069*	
C22	1.1358 (3)	0.5483 (3)	1.0052 (2)	0.0554 (10)	
H22	1.1864	0.5124	1.0337	0.067*	
C23	0.6453 (2)	0.7039 (2)	0.87754 (19)	0.0437 (8)	
H23	0.6978	0.6619	0.8860	0.052*	
C24	0.5551 (3)	0.6658 (3)	0.8443 (2)	0.0543 (10)	
H24	0.5481	0.6002	0.8314	0.065*	
C25	0.4776 (3)	0.7264 (3)	0.8312 (2)	0.0547 (10)	
H25	0.4173	0.7027	0.8081	0.066*	
C26	0.4886 (2)	0.8241 (3)	0.85246 (19)	0.0434 (8)	
C27	0.5820 (2)	0.8562 (2)	0.88643 (17)	0.0351 (7)	
C28	0.5960 (2)	0.9564 (2)	0.91083 (17)	0.0355 (7)	
C29	0.5157 (2)	1.0197 (2)	0.90190 (18)	0.0410 (8)	
C30	0.5319 (3)	1.1161 (3)	0.9279 (2)	0.0493 (9)	
H30	0.4809	1.1597	0.9235	0.059*	
C31	0.6228 (3)	1.1449 (3)	0.9596 (2)	0.0520 (10)	
H31	0.6343	1.2080	0.9784	0.062*	
C32	0.6986 (3)	1.0792 (2)	0.96363 (19)	0.0465 (9)	
H32	0.7608	1.1011	0.9828	0.056*	
C33	0.4103 (2)	0.8913 (3)	0.8426 (2)	0.0529 (10)	
H33	0.3492	0.8703	0.8190	0.063*	
C34	0.4227 (2)	0.9842 (3)	0.8666 (2)	0.0512 (9)	
H34	0.3700	1.0260	0.8602	0.061*	

### Atomic displacement parameters (Å^2^)

**Table:**

	*U*^11^	*U*^22^	*U*^33^	*U*^12^	*U*^13^	*U*^23^
La1	0.02833 (10)	0.02659 (10)	0.03019 (10)	0.00055 (9)	−0.00011 (7)	−0.00327 (8)
N1	0.0487 (17)	0.0416 (16)	0.0405 (16)	−0.0021 (14)	0.0130 (13)	−0.0099 (14)
N2	0.0389 (15)	0.0313 (14)	0.0356 (15)	−0.0021 (12)	0.0007 (12)	−0.0046 (12)
N3	0.0357 (15)	0.0316 (14)	0.0403 (16)	0.0000 (12)	−0.0008 (12)	−0.0019 (12)
N4	0.0377 (16)	0.0355 (15)	0.0399 (16)	−0.0008 (13)	0.0050 (12)	0.0019 (12)
N5	0.0376 (16)	0.0340 (15)	0.0389 (16)	0.0039 (12)	0.0010 (12)	0.0000 (12)
N6	0.0374 (16)	0.0448 (18)	0.0440 (18)	0.0005 (14)	0.0011 (13)	0.0050 (15)
N7	0.0520 (19)	0.052 (2)	0.045 (2)	−0.0089 (16)	0.0103 (15)	0.0023 (16)
O1	0.0377 (13)	0.0455 (13)	0.0386 (13)	−0.0086 (11)	0.0051 (10)	−0.0102 (10)
O2	0.0406 (13)	0.0418 (13)	0.0416 (13)	−0.0028 (11)	0.0061 (10)	−0.0147 (11)
O3	0.0758 (19)	0.0630 (18)	0.0515 (17)	−0.0085 (15)	−0.0001 (14)	−0.0028 (14)
O4	0.0558 (15)	0.0388 (13)	0.0436 (14)	−0.0127 (12)	−0.0044 (11)	0.0069 (11)
O5	0.0643 (17)	0.0401 (14)	0.0546 (16)	−0.0048 (12)	0.0028 (13)	−0.0074 (12)
O6	0.0721 (18)	0.0614 (17)	0.0509 (16)	0.0021 (14)	0.0088 (13)	0.0221 (14)
O7	0.0567 (16)	0.0445 (14)	0.0503 (15)	−0.0004 (12)	0.0025 (12)	−0.0050 (12)
O8	0.094 (2)	0.0383 (15)	0.0581 (17)	0.0041 (14)	0.0289 (14)	0.0002 (12)
O9	0.096 (2)	0.077 (2)	0.0511 (17)	−0.0066 (17)	0.0107 (15)	0.0189 (15)
C1	0.0351 (18)	0.0268 (16)	0.0341 (18)	0.0027 (14)	0.0024 (14)	−0.0043 (14)
C2	0.049 (2)	0.041 (2)	0.046 (2)	−0.0068 (17)	0.0148 (16)	−0.0127 (16)
C3	0.045 (2)	0.050 (2)	0.052 (2)	−0.0102 (18)	0.0189 (18)	−0.0066 (19)
C4	0.074 (3)	0.042 (2)	0.060 (2)	−0.006 (2)	0.020 (2)	−0.0070 (19)
C5	0.054 (2)	0.0401 (19)	0.045 (2)	0.0013 (18)	0.0157 (17)	−0.0090 (17)
C6	0.065 (3)	0.093 (3)	0.060 (3)	0.005 (2)	0.025 (2)	0.008 (2)
C7	0.111 (4)	0.106 (4)	0.057 (3)	0.022 (3)	0.033 (3)	0.019 (3)
C8	0.110 (4)	0.071 (3)	0.051 (3)	0.022 (3)	0.002 (3)	−0.003 (2)
C9	0.074 (3)	0.064 (3)	0.066 (3)	0.005 (2)	−0.005 (2)	−0.017 (2)
C10	0.062 (3)	0.053 (2)	0.053 (2)	−0.006 (2)	0.0125 (19)	−0.0110 (19)
C11	0.042 (2)	0.043 (2)	0.038 (2)	−0.0083 (16)	0.0018 (15)	−0.0070 (16)
C12	0.057 (2)	0.046 (2)	0.043 (2)	−0.0117 (18)	0.0143 (18)	−0.0166 (17)
C13	0.071 (3)	0.035 (2)	0.059 (2)	−0.0058 (19)	0.033 (2)	−0.0110 (18)
C14	0.051 (2)	0.0297 (18)	0.051 (2)	0.0005 (16)	0.0187 (17)	0.0004 (16)
C15	0.0376 (18)	0.0296 (17)	0.0377 (18)	−0.0029 (14)	0.0105 (14)	0.0017 (14)
C16	0.0351 (18)	0.0300 (17)	0.0390 (19)	−0.0004 (14)	0.0072 (14)	0.0041 (14)
C17	0.0396 (19)	0.043 (2)	0.053 (2)	0.0018 (17)	0.0096 (16)	0.0114 (18)
C18	0.045 (2)	0.055 (2)	0.057 (2)	0.0069 (19)	−0.0048 (18)	0.016 (2)
C19	0.056 (2)	0.063 (3)	0.047 (2)	0.002 (2)	−0.0134 (19)	0.0025 (19)
C20	0.050 (2)	0.044 (2)	0.044 (2)	0.0024 (17)	−0.0078 (17)	−0.0058 (17)
C21	0.070 (3)	0.035 (2)	0.072 (3)	0.0145 (19)	0.029 (2)	0.0054 (19)
C22	0.055 (2)	0.044 (2)	0.069 (3)	0.0198 (19)	0.015 (2)	0.017 (2)
C23	0.041 (2)	0.0371 (19)	0.053 (2)	−0.0028 (16)	0.0078 (16)	−0.0018 (17)
C24	0.055 (2)	0.044 (2)	0.063 (3)	−0.0121 (19)	0.0084 (19)	−0.0058 (19)
C25	0.042 (2)	0.059 (2)	0.060 (2)	−0.0133 (19)	0.0020 (18)	−0.002 (2)
C26	0.0361 (19)	0.050 (2)	0.044 (2)	−0.0025 (17)	0.0047 (15)	0.0067 (17)
C27	0.0309 (17)	0.0405 (19)	0.0336 (17)	0.0022 (15)	0.0051 (13)	0.0067 (14)
C28	0.0360 (18)	0.0400 (19)	0.0304 (17)	0.0019 (15)	0.0052 (14)	0.0054 (14)
C29	0.040 (2)	0.047 (2)	0.0370 (19)	0.0089 (17)	0.0114 (15)	0.0121 (16)
C30	0.053 (2)	0.049 (2)	0.046 (2)	0.0220 (19)	0.0081 (17)	0.0064 (18)
C31	0.067 (3)	0.040 (2)	0.045 (2)	0.0146 (19)	−0.0013 (18)	−0.0008 (16)
C32	0.050 (2)	0.041 (2)	0.044 (2)	0.0034 (17)	−0.0029 (17)	−0.0035 (17)
C33	0.0310 (19)	0.066 (3)	0.061 (2)	−0.0021 (18)	0.0061 (16)	0.011 (2)
C34	0.035 (2)	0.061 (3)	0.058 (2)	0.0111 (19)	0.0106 (17)	0.019 (2)

### Geometric parameters (Å, °)

**Table:**

La1—O2^i^	2.376 (2)	C8—H8	0.9300
La1—O1	2.492 (2)	C9—C10	1.376 (5)
La1—O4	2.593 (2)	C9—H9	0.9300
La1—O8	2.610 (2)	C10—H10	0.9300
La1—N3	2.680 (2)	C11—C12	1.387 (5)
La1—N2	2.709 (2)	C11—H11	0.9300
La1—O5	2.715 (2)	C12—C13	1.356 (5)
La1—N5	2.740 (2)	C12—H12	0.9300
La1—O7	2.754 (2)	C13—C14	1.402 (5)
La1—N4	2.788 (3)	C13—H13	0.9300
N1—C3	1.363 (4)	C14—C15	1.408 (4)
N1—C5	1.433 (4)	C14—C21	1.425 (5)
N1—C2	1.455 (4)	C15—C16	1.441 (4)
N2—C11	1.327 (4)	C16—C17	1.400 (4)
N2—C15	1.362 (4)	C17—C18	1.403 (5)
N3—C20	1.321 (4)	C17—C22	1.429 (5)
N3—C16	1.369 (4)	C18—C19	1.357 (5)
N4—C23	1.325 (4)	C18—H18	0.9300
N4—C27	1.364 (4)	C19—C20	1.391 (5)
N5—C32	1.330 (4)	C19—H19	0.9300
N5—C28	1.357 (4)	C20—H20	0.9300
N6—O6	1.224 (3)	C21—C22	1.345 (5)
N6—O5	1.252 (3)	C21—H21	0.9300
N6—O4	1.268 (3)	C22—H22	0.9300
N7—O9	1.229 (3)	C23—C24	1.400 (4)
N7—O8	1.248 (4)	C23—H23	0.9300
N7—O7	1.253 (3)	C24—C25	1.361 (5)
O1—C1	1.239 (3)	C24—H24	0.9300
O2—C1	1.261 (3)	C25—C26	1.393 (5)
O2—La1^i^	2.376 (2)	C25—H25	0.9300
O3—C3	1.221 (4)	C26—C27	1.411 (4)
C1—C2	1.518 (4)	C26—C33	1.427 (5)
C2—H2A	0.9700	C27—C28	1.444 (4)
C2—H2B	0.9700	C28—C29	1.414 (4)
C3—C4	1.507 (5)	C29—C30	1.403 (5)
C4—H4A	0.9600	C29—C34	1.428 (5)
C4—H4B	0.9600	C30—C31	1.358 (5)
C4—H4C	0.9600	C30—H30	0.9300
C5—C6	1.370 (5)	C31—C32	1.392 (5)
C5—C10	1.376 (5)	C31—H31	0.9300
C6—C7	1.387 (6)	C32—H32	0.9300
C6—H6	0.9300	C33—C34	1.344 (5)
C7—C8	1.365 (6)	C33—H33	0.9300
C7—H7	0.9300	C34—H34	0.9300
C8—C9	1.353 (6)		
			
O2^i^—La1—O1	83.13 (7)	C6—C5—C10	119.7 (4)
O2^i^—La1—O4	125.60 (7)	C6—C5—N1	119.8 (3)
O1—La1—O4	74.74 (7)	C10—C5—N1	120.5 (3)
O2^i^—La1—O8	70.80 (8)	C5—C6—C7	119.6 (4)
O1—La1—O8	137.26 (8)	C5—C6—H6	120.2
O4—La1—O8	147.97 (8)	C7—C6—H6	120.2
O2^i^—La1—N3	84.08 (7)	C8—C7—C6	120.2 (4)
O1—La1—N3	69.20 (7)	C8—C7—H7	119.9
O4—La1—N3	129.44 (8)	C6—C7—H7	119.9
O8—La1—N3	74.80 (8)	C9—C8—C7	119.9 (4)
O2^i^—La1—N2	141.31 (7)	C9—C8—H8	120.0
O1—La1—N2	69.05 (7)	C7—C8—H8	120.0
O4—La1—N2	73.23 (7)	C8—C9—C10	120.7 (4)
O8—La1—N2	112.44 (8)	C8—C9—H9	119.6
N3—La1—N2	61.51 (7)	C10—C9—H9	119.6
O2^i^—La1—O5	77.98 (7)	C5—C10—C9	119.8 (4)
O1—La1—O5	65.36 (7)	C5—C10—H10	120.1
O4—La1—O5	47.63 (7)	C9—C10—H10	120.1
O8—La1—O5	135.76 (8)	N2—C11—C12	124.2 (3)
N3—La1—O5	132.60 (8)	N2—C11—H11	117.9
N2—La1—O5	111.57 (7)	C12—C11—H11	117.9
O2^i^—La1—N5	77.10 (7)	C13—C12—C11	118.6 (3)
O1—La1—N5	129.03 (7)	C13—C12—H12	120.7
O4—La1—N5	79.69 (7)	C11—C12—H12	120.7
O8—La1—N5	78.07 (8)	C12—C13—C14	119.9 (3)
N3—La1—N5	150.86 (8)	C12—C13—H13	120.1
N2—La1—N5	141.46 (7)	C14—C13—H13	120.1
O5—La1—N5	64.76 (7)	C13—C14—C15	117.8 (3)
O2^i^—La1—O7	113.62 (7)	C13—C14—C21	122.1 (3)
O1—La1—O7	125.74 (7)	C15—C14—C21	120.1 (3)
O4—La1—O7	119.74 (7)	N2—C15—C14	122.0 (3)
O8—La1—O7	46.86 (7)	N2—C15—C16	119.2 (3)
N3—La1—O7	62.44 (7)	C14—C15—C16	118.8 (3)
N2—La1—O7	67.34 (7)	N3—C16—C17	122.2 (3)
O5—La1—O7	163.54 (7)	N3—C16—C15	118.4 (3)
N5—La1—O7	105.22 (7)	C17—C16—C15	119.4 (3)
O2^i^—La1—N4	132.04 (7)	C16—C17—C18	117.6 (3)
O1—La1—N4	139.19 (7)	C16—C17—C22	120.0 (3)
O4—La1—N4	67.69 (7)	C18—C17—C22	122.3 (3)
O8—La1—N4	81.15 (8)	C19—C18—C17	119.8 (3)
N3—La1—N4	125.42 (8)	C19—C18—H18	120.1
N2—La1—N4	85.05 (8)	C17—C18—H18	120.1
O5—La1—N4	98.33 (7)	C18—C19—C20	119.2 (3)
N5—La1—N4	59.17 (8)	C18—C19—H19	120.4
O7—La1—N4	65.28 (7)	C20—C19—H19	120.4
C3—N1—C5	123.5 (3)	N3—C20—C19	123.4 (3)
C3—N1—C2	117.3 (3)	N3—C20—H20	118.3
C5—N1—C2	119.0 (3)	C19—C20—H20	118.3
C11—N2—C15	117.4 (3)	C22—C21—C14	120.9 (3)
C11—N2—La1	122.7 (2)	C22—C21—H21	119.6
C15—N2—La1	119.86 (18)	C14—C21—H21	119.6
C20—N3—C16	117.8 (3)	C21—C22—C17	120.7 (3)
C20—N3—La1	121.1 (2)	C21—C22—H22	119.6
C16—N3—La1	121.06 (19)	C17—C22—H22	119.6
C23—N4—C27	117.0 (3)	N4—C23—C24	123.8 (3)
C23—N4—La1	121.8 (2)	N4—C23—H23	118.1
C27—N4—La1	120.83 (19)	C24—C23—H23	118.1
C32—N5—C28	117.8 (3)	C25—C24—C23	118.8 (3)
C32—N5—La1	119.2 (2)	C25—C24—H24	120.6
C28—N5—La1	122.8 (2)	C23—C24—H24	120.6
O6—N6—O5	122.4 (3)	C24—C25—C26	120.0 (3)
O6—N6—O4	120.8 (3)	C24—C25—H25	120.0
O5—N6—O4	116.8 (3)	C26—C25—H25	120.0
O9—N7—O8	121.4 (3)	C25—C26—C27	117.5 (3)
O9—N7—O7	121.1 (3)	C25—C26—C33	123.1 (3)
O8—N7—O7	117.4 (3)	C27—C26—C33	119.4 (3)
C1—O1—La1	138.33 (19)	N4—C27—C26	122.8 (3)
C1—O2—La1^i^	161.3 (2)	N4—C27—C28	118.0 (3)
N6—O4—La1	100.05 (18)	C26—C27—C28	119.2 (3)
N6—O5—La1	94.59 (18)	N5—C28—C29	122.2 (3)
N7—O7—La1	92.03 (18)	N5—C28—C27	118.4 (3)
N7—O8—La1	99.1 (2)	C29—C28—C27	119.4 (3)
O1—C1—O2	125.5 (3)	C30—C29—C28	117.7 (3)
O1—C1—C2	118.8 (3)	C30—C29—C34	122.8 (3)
O2—C1—C2	115.7 (3)	C28—C29—C34	119.5 (3)
N1—C2—C1	115.0 (3)	C31—C30—C29	119.4 (3)
N1—C2—H2A	108.5	C31—C30—H30	120.3
C1—C2—H2A	108.5	C29—C30—H30	120.3
N1—C2—H2B	108.5	C30—C31—C32	119.4 (3)
C1—C2—H2B	108.5	C30—C31—H31	120.3
H2A—C2—H2B	107.5	C32—C31—H31	120.3
O3—C3—N1	120.5 (3)	N5—C32—C31	123.4 (3)
O3—C3—C4	121.9 (4)	N5—C32—H32	118.3
N1—C3—C4	117.5 (3)	C31—C32—H32	118.3
C3—C4—H4A	109.5	C34—C33—C26	121.7 (3)
C3—C4—H4B	109.5	C34—C33—H33	119.2
H4A—C4—H4B	109.5	C26—C33—H33	119.2
C3—C4—H4C	109.5	C33—C34—C29	120.9 (3)
H4A—C4—H4C	109.5	C33—C34—H34	119.6
H4B—C4—H4C	109.5	C29—C34—H34	119.6
			
O2^i^—La1—N2—C11	−147.4 (2)	N5—La1—O7—N7	−68.74 (19)
O1—La1—N2—C11	−100.4 (2)	N4—La1—O7—N7	−113.51 (19)
O4—La1—N2—C11	−20.7 (2)	O9—N7—O8—La1	154.8 (3)
O8—La1—N2—C11	125.7 (2)	O7—N7—O8—La1	−22.5 (3)
N3—La1—N2—C11	−177.4 (3)	O2^i^—La1—O8—N7	−143.0 (2)
O5—La1—N2—C11	−49.6 (2)	O1—La1—O8—N7	−87.3 (2)
N5—La1—N2—C11	26.6 (3)	O4—La1—O8—N7	89.7 (2)
O7—La1—N2—C11	112.7 (2)	N3—La1—O8—N7	−54.1 (2)
N4—La1—N2—C11	47.4 (2)	N2—La1—O8—N7	−4.4 (2)
O2^i^—La1—N2—C15	30.4 (3)	O5—La1—O8—N7	169.36 (18)
O1—La1—N2—C15	77.3 (2)	N5—La1—O8—N7	136.7 (2)
O4—La1—N2—C15	157.0 (2)	O7—La1—O8—N7	12.21 (18)
O8—La1—N2—C15	−56.6 (2)	N4—La1—O8—N7	76.5 (2)
N3—La1—N2—C15	0.3 (2)	La1—O1—C1—O2	3.0 (5)
O5—La1—N2—C15	128.1 (2)	La1—O1—C1—C2	−175.1 (2)
N5—La1—N2—C15	−155.73 (19)	La1^i^—O2—C1—O1	−135.8 (5)
O7—La1—N2—C15	−69.6 (2)	La1^i^—O2—C1—C2	42.3 (7)
N4—La1—N2—C15	−134.9 (2)	C3—N1—C2—C1	−71.2 (4)
O2^i^—La1—N3—C20	18.8 (2)	C5—N1—C2—C1	103.4 (3)
O1—La1—N3—C20	103.7 (3)	O1—C1—C2—N1	−26.0 (4)
O4—La1—N3—C20	151.1 (2)	O2—C1—C2—N1	155.8 (3)
O8—La1—N3—C20	−52.8 (2)	C5—N1—C3—O3	−175.7 (3)
N2—La1—N3—C20	−179.5 (3)	C2—N1—C3—O3	−1.3 (5)
O5—La1—N3—C20	86.5 (3)	C5—N1—C3—C4	6.1 (5)
N5—La1—N3—C20	−30.9 (3)	C2—N1—C3—C4	−179.5 (3)
O7—La1—N3—C20	−101.7 (3)	C3—N1—C5—C6	−91.5 (4)
N4—La1—N3—C20	−119.9 (2)	C2—N1—C5—C6	94.2 (4)
O2^i^—La1—N3—C16	−162.5 (2)	C3—N1—C5—C10	88.9 (4)
O1—La1—N3—C16	−77.6 (2)	C2—N1—C5—C10	−85.5 (4)
O4—La1—N3—C16	−30.2 (3)	C10—C5—C6—C7	−2.5 (6)
O8—La1—N3—C16	125.8 (2)	N1—C5—C6—C7	177.8 (4)
N2—La1—N3—C16	−0.8 (2)	C5—C6—C7—C8	1.8 (7)
O5—La1—N3—C16	−94.8 (2)	C6—C7—C8—C9	0.5 (7)
N5—La1—N3—C16	147.8 (2)	C7—C8—C9—C10	−1.9 (7)
O7—La1—N3—C16	77.0 (2)	C6—C5—C10—C9	1.1 (6)
N4—La1—N3—C16	58.7 (2)	N1—C5—C10—C9	−179.3 (3)
O2^i^—La1—N4—C23	−151.3 (2)	C8—C9—C10—C5	1.1 (6)
O1—La1—N4—C23	65.8 (3)	C15—N2—C11—C12	1.1 (5)
O4—La1—N4—C23	90.2 (2)	La1—N2—C11—C12	178.9 (2)
O8—La1—N4—C23	−97.3 (2)	N2—C11—C12—C13	0.5 (5)
N3—La1—N4—C23	−33.2 (3)	C11—C12—C13—C14	−1.3 (5)
N2—La1—N4—C23	16.3 (2)	C12—C13—C14—C15	0.4 (5)
O5—La1—N4—C23	127.4 (2)	C12—C13—C14—C21	−179.6 (3)
N5—La1—N4—C23	−178.7 (3)	C11—N2—C15—C14	−2.0 (4)
O7—La1—N4—C23	−51.0 (2)	La1—N2—C15—C14	−179.9 (2)
O2^i^—La1—N4—C27	35.3 (3)	C11—N2—C15—C16	178.1 (3)
O1—La1—N4—C27	−107.6 (2)	La1—N2—C15—C16	0.2 (4)
O4—La1—N4—C27	−83.2 (2)	C13—C14—C15—N2	1.3 (5)
O8—La1—N4—C27	89.3 (2)	C21—C14—C15—N2	−178.7 (3)
N3—La1—N4—C27	153.4 (2)	C13—C14—C15—C16	−178.8 (3)
N2—La1—N4—C27	−157.1 (2)	C21—C14—C15—C16	1.2 (5)
O5—La1—N4—C27	−45.9 (2)	C20—N3—C16—C17	0.1 (5)
N5—La1—N4—C27	8.0 (2)	La1—N3—C16—C17	−178.6 (2)
O7—La1—N4—C27	135.6 (2)	C20—N3—C16—C15	−179.9 (3)
O2^i^—La1—N5—C32	19.8 (2)	La1—N3—C16—C15	1.3 (4)
O1—La1—N5—C32	−50.2 (3)	N2—C15—C16—N3	−1.0 (4)
O4—La1—N5—C32	−110.7 (2)	C14—C15—C16—N3	179.1 (3)
O8—La1—N5—C32	92.5 (2)	N2—C15—C16—C17	178.9 (3)
N3—La1—N5—C32	70.9 (3)	C14—C15—C16—C17	−1.0 (4)
N2—La1—N5—C32	−156.3 (2)	N3—C16—C17—C18	1.1 (5)
O5—La1—N5—C32	−62.9 (2)	C15—C16—C17—C18	−178.8 (3)
O7—La1—N5—C32	131.1 (2)	N3—C16—C17—C22	179.9 (3)
N4—La1—N5—C32	179.2 (3)	C15—C16—C17—C22	0.0 (5)
O2^i^—La1—N5—C28	−166.6 (2)	C16—C17—C18—C19	−0.9 (5)
O1—La1—N5—C28	123.5 (2)	C22—C17—C18—C19	−179.7 (4)
O4—La1—N5—C28	62.9 (2)	C17—C18—C19—C20	−0.5 (6)
O8—La1—N5—C28	−93.9 (2)	C16—N3—C20—C19	−1.6 (5)
N3—La1—N5—C28	−115.5 (2)	La1—N3—C20—C19	177.1 (3)
N2—La1—N5—C28	17.3 (3)	C18—C19—C20—N3	1.8 (6)
O5—La1—N5—C28	110.8 (2)	C13—C14—C21—C22	179.7 (3)
O7—La1—N5—C28	−55.3 (2)	C15—C14—C21—C22	−0.4 (5)
N4—La1—N5—C28	−7.1 (2)	C14—C21—C22—C17	−0.7 (6)
O2^i^—La1—O1—C1	11.0 (3)	C16—C17—C22—C21	0.9 (5)
O4—La1—O1—C1	140.9 (3)	C18—C17—C22—C21	179.7 (3)
O8—La1—O1—C1	−40.8 (3)	C27—N4—C23—C24	1.5 (5)
N3—La1—O1—C1	−75.2 (3)	La1—N4—C23—C24	−172.2 (3)
N2—La1—O1—C1	−141.6 (3)	N4—C23—C24—C25	0.3 (5)
O5—La1—O1—C1	90.9 (3)	C23—C24—C25—C26	−1.4 (5)
N5—La1—O1—C1	78.2 (3)	C24—C25—C26—C27	0.7 (5)
O7—La1—O1—C1	−103.2 (3)	C24—C25—C26—C33	−178.2 (3)
N4—La1—O1—C1	164.2 (3)	C23—N4—C27—C26	−2.2 (4)
O6—N6—O4—La1	170.4 (2)	La1—N4—C27—C26	171.5 (2)
O5—N6—O4—La1	−10.1 (3)	C23—N4—C27—C28	177.6 (3)
O2^i^—La1—O4—N6	4.9 (2)	La1—N4—C27—C28	−8.7 (4)
O1—La1—O4—N6	−64.73 (18)	C25—C26—C27—N4	1.1 (5)
O8—La1—O4—N6	117.36 (19)	C33—C26—C27—N4	−179.9 (3)
N3—La1—O4—N6	−110.21 (18)	C25—C26—C27—C28	−178.7 (3)
N2—La1—O4—N6	−136.97 (19)	C33—C26—C27—C28	0.3 (5)
O5—La1—O4—N6	5.63 (16)	C32—N5—C28—C29	0.1 (4)
N5—La1—O4—N6	70.76 (18)	La1—N5—C28—C29	−173.6 (2)
O7—La1—O4—N6	172.49 (17)	C32—N5—C28—C27	179.9 (3)
N4—La1—O4—N6	131.5 (2)	La1—N5—C28—C27	6.2 (4)
O6—N6—O5—La1	−170.9 (3)	N4—C27—C28—N5	1.8 (4)
O4—N6—O5—La1	9.6 (3)	C26—C27—C28—N5	−178.4 (3)
O2^i^—La1—O5—N6	173.77 (19)	N4—C27—C28—C29	−178.4 (3)
O1—La1—O5—N6	85.88 (18)	C26—C27—C28—C29	1.5 (4)
O4—La1—O5—N6	−5.63 (16)	N5—C28—C29—C30	−1.6 (5)
O8—La1—O5—N6	−140.71 (17)	C27—C28—C29—C30	178.6 (3)
N3—La1—O5—N6	103.60 (18)	N5—C28—C29—C34	177.9 (3)
N2—La1—O5—N6	33.07 (19)	C27—C28—C29—C34	−2.0 (4)
N5—La1—O5—N6	−104.96 (19)	C28—C29—C30—C31	0.6 (5)
O7—La1—O5—N6	−49.8 (3)	C34—C29—C30—C31	−178.8 (3)
N4—La1—O5—N6	−54.87 (18)	C29—C30—C31—C32	1.7 (5)
O9—N7—O7—La1	−156.3 (3)	C28—N5—C32—C31	2.5 (5)
O8—N7—O7—La1	21.0 (3)	La1—N5—C32—C31	176.4 (3)
O2^i^—La1—O7—N7	13.6 (2)	C30—C31—C32—N5	−3.4 (5)
O1—La1—O7—N7	112.44 (18)	C25—C26—C33—C34	177.3 (4)
O4—La1—O7—N7	−155.40 (17)	C27—C26—C33—C34	−1.6 (5)
O8—La1—O7—N7	−12.01 (18)	C26—C33—C34—C29	1.1 (5)
N3—La1—O7—N7	82.79 (19)	C30—C29—C34—C33	−179.9 (3)
N2—La1—O7—N7	151.4 (2)	C28—C29—C34—C33	0.7 (5)
O5—La1—O7—N7	−119.1 (3)		

Symmetry codes: (i) −*x*+2, −*y*+2, −*z*+2.
